# The microbiome and the hallmarks of cancer

**DOI:** 10.1371/journal.ppat.1006480

**Published:** 2017-09-21

**Authors:** Laura E. Fulbright, Melissa Ellermann, Janelle C. Arthur

**Affiliations:** 1 Department of Microbiology and Immunology, University of North Carolina at Chapel Hill, Chapel Hill, North Carolina, United States of America; 2 Center for Gastrointestinal Biology and Disease, University of North Carolina at Chapel Hill, Chapel Hill, North Carolina, United States of America; 3 Lineberger Comprehensive Cancer Center, University of North Carolina at Chapel Hill, Chapel Hill, North Carolina, United States of America; Tufts Univ School of Medicine, UNITED STATES

## Introduction

**Fig 1:**
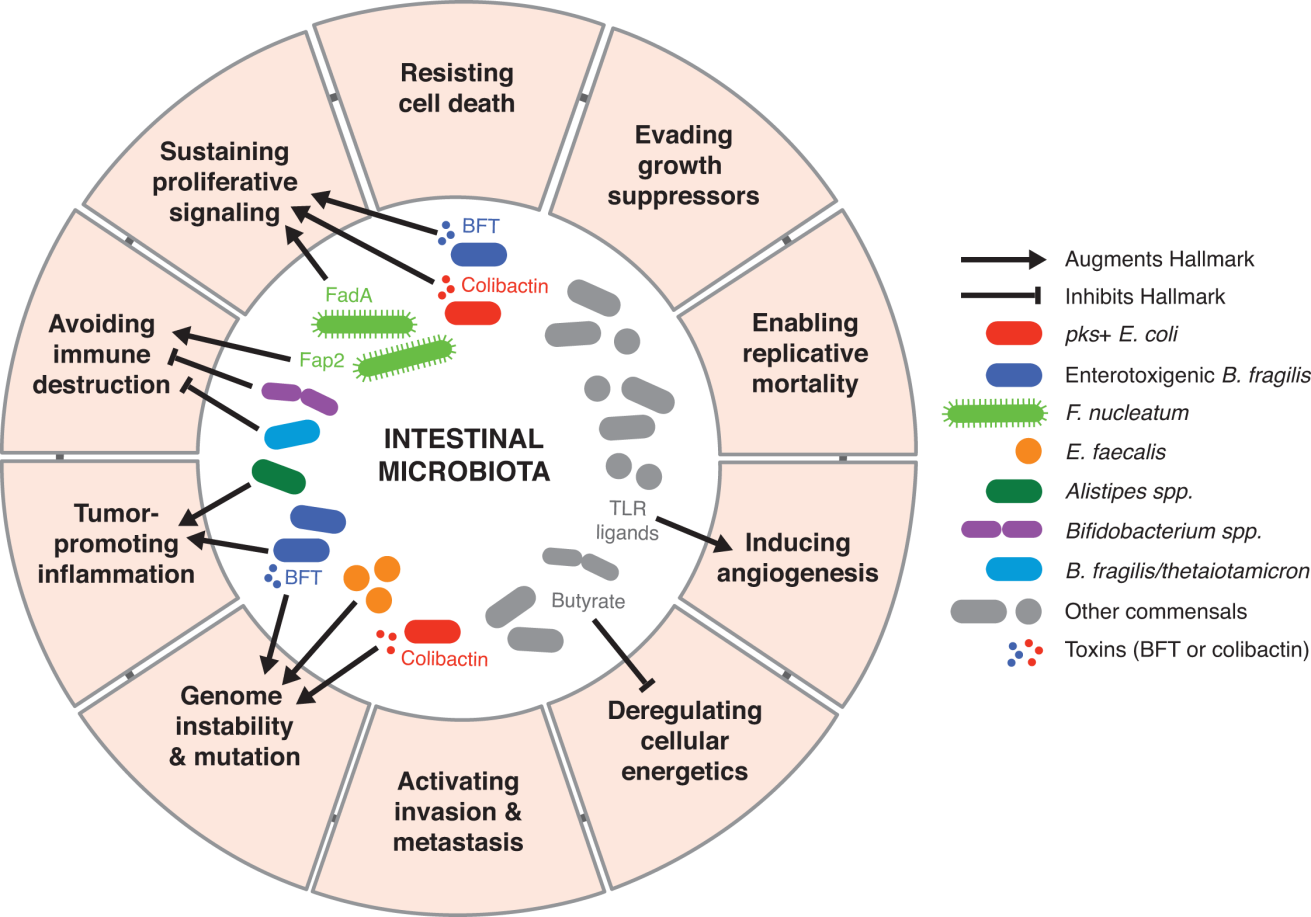
Microbial-derived signals modulate numerous hallmarks of cancer through diverse mechanisms.

The “Hallmarks of Cancer,” proposed by Hanahan and Weinburg in 2001 and updated in 2011, logically define how a normal cell progresses to a tumorigenic state within a complex neoplastic environment [1]. These hallmark capabilities have given us remarkable insight into the multistep changes that occur within the tissue microenvironment during cancer development. However, it has become well established that host-associated microbial communities, termed microbiota, also play integral roles in modulating various aspects of host physiology. This includes host processes such as cellular metabolism and immune function that become highly dysregulated during carcinogenesis. Perturbations to the microbiota also disrupt these homeostatic processes, promoting the development of numerous diseases including inflammatory bowel diseases (IBD) and colorectal cancer (CRC). *Helicobacter pylori* served as the initial link between bacteria and cancer, when it was discovered that infection predisposed humans to gastric cancer [2]. More recently, fast and inexpensive next-generation sequencing methods combined with research initiatives to support multi-investigator research teams (for example, the National Institutes of Health (NIH)-funded Human Microbiome Project) have revolutionized our understanding of the microbiota and human disease. In parallel, animal models have demonstrated a causal relationship between particular microbes and cancer development through fecal transplants from cancer-bearing mice or inoculation of cancer-associated microbes into formerly germ-free mice. Together, these studies have shown that our resident microbes likely influence the initiation and progression of tumorigenesis by modulating most, if not all, established host factors that comprise the hallmarks of cancer. Further knowledge defining how the microbiota modulates host physiology and disease pathogenesis, particularly in the context of cancer, will provide a framework for the holobiont concept of cancer development and enable the identification of novel microbial targets for preventative and therapeutic strategies. This review will explore how specific members of the microbiota, summarized in Fig 1 and Table 1, influence the hallmarks of cancer.

**Table 1:** Members of the intestinal microbiota associated with cancer development and resistance.

Intestinal bacteria	Bacterial mechanism	Hallmark affected	Mouse models	References
enterotoxigenic *Bacteroides fragilis* (ETBF)	*B*. *fragilis* toxin (BFT)	sustaining proliferative signaling	WT mice	[3]
		genome instability and mutations	*Apc*^*Min/+*^	[21]
	unknown mechanism	tumor-promoting inflammation	*Apc*^*Min/+*^	[10]
*Fusobacterium nucleatum*	FadA adhesin	sustaining proliferative signaling	xenograft model	[4]
	Fap2 adhesin	avoiding immune destruction	*Apc*^*Min/+*^	[14] [13]
*pks*+ *Escherichia coli*	colibactin	genome instability and mutations	in vitro cellular assays	[19]
			AOM/*Il10*^-/-^	[20]
		sustaining proliferative signaling	AOM/DSS xenograft model	[5]
*Enterococcus faecalis*	unknown mechanism	genome instability and mutations	allograft model	[22]
*Alistipes spp*.	unknown mechanism	tumor-promoting inflammation	*Il10*^*-/-*^ *Lcn2*^*-/-*^	[12]
*Bifidobacterium spp*.	unknown mechanism	inhibits avoiding immune destruction	subcutaneous B16.SIY melanoma	[15]
*Bacteroides thetaiotamicron* and *B*. *fragilis*	unknown mechanism	inhibits avoiding immune destruction	MCA205 sarcoma, Ret melanoma, and MC38 CRC xenograft	[16]

**Abbreviations:** AOM, azoxymethane; Apc, adenomatosis polyposis coli; CRC, colorectal cancer; DSS, dextran sodium sulfate; *Il10*, interleukin 10; *Lcn2*, lipocalin2; Min, multiple intestinal neoplasia

### How does the microbiota influence cellular proliferation and host cellular energetics?

Normal tissues tightly regulate growth-promoting and death-inducing signals to maintain homeostatic cell densities, tissue architecture, and function. Dysregulation of these signaling pathways can lead to sustained cellular proliferation. The intercellular adhesion molecule, E-cadherin, is a common target engaged by intestinal bacteria that promotes epithelial proliferation by activating the Wnt/ß-catenin pathway. For example, enterotoxigenic *Bacteroides fragilis* (ETBF), resident among the microbiota of some individuals, secretes *B*. *fragilis* toxin (BFT) that promotes cleavage of E-cadherin [3]. This enables the nuclear translocation of ß-catenin, subsequent transcription of proto-oncogene c-Myc, and colonic epithelial hyperplasia [3]. Through a similar mechanism, *Fusobacterium nucleatum* enhances epithelial proliferation through engagement of its adhesin FadA with E-cadherin [4]. Neutralizing FadA abrogated the tumor-promoting activities of *F*. *nucleatum* in a murine xenograft cancer model [4], demonstrating the potential of targeting bacterial interactions with E-cadherin as a novel strategy in mitigating cancer progression. Taken together, these studies demonstrate that the microbiota can be a source of activating signals for aberrant epithelial proliferation as an initiating step in cancer development.

Cellular senescence—when cells cease to divide—is often considered a barrier for proliferation. However, senescent cells secrete growth factors that enable tumor growth, and intestinal bacteria may induce this pathway to malignancy. Colibactin-producing (*pks+) Escherichia coli* induce a senescence-associated secretory phenotype (SASP) in which senescent cells secrete growth factors that stimulate epithelial proliferation and enhance tumor growth [5]. Thus, microbial-induced cellular senescence and bystander proliferation provide additional mechanisms by which malignancy can arise from host–microbial interactions.

Perturbations to the local metabolic environment can also favor or inhibit sustained cancer cell proliferation and tumorigenesis. For example, the microbial metabolome has long been established as a modulator of host cellular metabolism. Short-chain fatty acids such as butyrate are generated through microbial fermentation of dietary fibers and are a preferred primary energy source for colonocytes. In contrast, cancer cells preferentially utilize glucose as a carbon source through glycolysis—a phenomenon known as the Warburg effect. Butyrate not only exerts an anticancer effect by starving cancer cells, but impaired butyrate metabolism increases intracellular concentrations of butyrate, which acts as a histone deacetylase inhibitor and promotes apoptosis and inhibition of cellular proliferation through epigenetic modifications [6]. Given the complexity of the microbial metabolome, it will be important to broaden our investigation beyond individual metabolites and consider the impact of the metabolome as a whole on cellular energetics and other hallmarks of cancer.

### How does the microbiota shape the local tumor microenvironment?

The microbiota influences cancer development by modulating the local tumor microenvironment through its effects on tissue remodeling and mucosal immunity. Angiogenesis, one aspect of tissue remodeling that occurs during tumorigenesis, enables adequate blood flow, which is integral for tumor persistence and proliferation. Although direct links between endogenous bacteria and tumor-associated angiogenesis have not been reported, the microbiota is required for normal development of the vasculature within the intestines [7]. Moreover, in the context of infection, microbial products such as lipopolysaccharide engage with Toll-like receptors to promote angiogenesis, an effect that is augmented by damage-associated molecular patterns that may also be present within the tumor microenvironment [8]. Further studies will determine whether specific microbes influence angiogenesis and tumor-associated remodeling of the vasculature.

The close proximity of the microbiota and mucosal immune system also provides the potential for endogenous bacteria to impact the tumor microenvironment by stimulating a variety of protumorigenic immune responses. T-helper-17 (Th17) immunity is generally protumorigenic, associated with worse prognosis in CRC, and driven by microbes and microbial products [9]. Colonization of tumor-susceptible adenomatosis polyposis coli–multiple intestinal neoplasia (*Apc*^*Min/+*^) mice with ETBF enhances Th17-driven inflammation and colonic tumor development [10] [11]. Blocking the interleukin-(IL)-17 signaling axis reduces downstream signal transducer and activator of transcription factor 3 (STAT3) signaling in tumor and nontumor cells, thus preventing inflammation and tumorigenesis [10] [11]. Similarly, the carcinogenic potential of the intestinal commensal *Alistipes* is associated with enhanced IL-6 production, STAT3 activation, epithelial hyperplasia, and epithelial barrier dysfunction [12]. Thus, specific members of the microbiota stimulate Th17-driven inflammation and aid in establishing a tumor-permissive inflammatory environment.

While Th17 immune responses promote tumor development, others involving cytotoxic immune cells are essential for identifying and destroying precancerous and malignant cells. *F*. *nucleatum* dampens this arm of cancer immunity through 2 distinct mechanisms to enable tumor progression and persistence. *F*. *nucleatum* utilizes its Fap2 adhesion to silence the tumor-killing capabilities of cytotoxic immune cells through direct interaction with the immune inhibitory receptor T-cell immunoreceptor with immunoglobulin and immunoreceptor tyrosine-based inhibitory motif domains (TIGIT) [13]. *F*. *nucleatum* abundance is also correlated in clinical and animal studies with an enrichment of myeloid-derived suppressor cells and tumor-associated macrophages, both of which inhibit antitumor T-cell responses [14].

While some resident intestinal bacteria inhibit antitumor immunity, others stimulate antitumor immunity and potentiate cancer immunotherapy. *Bifidobacterium* augments dendritic cell function and subsequent tumor-killing capabilities of cytotoxic T cells, which correspond with reduced growth of subcutaneous melanoma xenograft models in mice [15]. *Bifidobacterium* administration in combination with the established anticancer immunotherapeutic programmed death-ligand 1 (PD-L1) blockade nearly abolished tumor growth [15]. Similarly, *Bacteroides thetaiotamicron* and nontoxigenic *B*. *fragilis* improve the efficacy of an anti–cytotoxic T-lymphocyte-associated protein 4 (CTLA4) immunotherapeutic tested in 3 cancer xenograft mouse models. This is achieved by augmenting antitumor cytotoxic T-cell immunity and is associated with T-cell responses specific for *B*. *thetaiotamicron* or *B*. *fragilis* [16]. While *B*. *fragilis* polysaccharides can enhance antitumor immunity [16], the specific *B*. *fragilis* polysaccharide A (PSA) promotes an anti-inflammatory state in the intestine by fine-tuning the balance of effector and regulatory T cells [17] [18]. However, it remains unclear whether the anti-inflammatory effects of PSA impact cancer development and the efficacy of cancer immunotherapies. Together, these findings introduce the exciting prospect of manipulating the microbiota as a means of not only modulating cancer-associated tissue remodeling and immunity but also enhancing the efficacy of established anticancer therapies.

### Does the microbiota promote genome instability and mutations?

The breakdown of genome maintenance within the host, whether through DNA damage accumulation or failure to properly segregate chromosomes, allows premalignant and malignant cells to both retain and accelerate the rate of mutations. Several gut microbes are a potential source of DNA mutagens. In vitro studies first demonstrated that *pks+ E*. *coli* induce DNA double-strand breaks, aneuploidy, cell-cycle arrest, and improper cellular division [19]. Multiple animal models of CRC have demonstrated that *pks+ E*. *coli* promote DNA damage in vivo, yet inflammation remains unaffected and unlikely to be a driving force behind this damage [20] [5]. In contrast, other resident microbes can induce DNA damage by promoting inflammation and a pro-oxidant microenvironment. ETBF induces colonic epithelial expression of spermine oxidase (SMO), an enzyme that generates the DNA-damaging agent peroxide. Inhibition of SMO prevents ETBF-induced DNA damage, which corresponds with a decrease in ETBF-induced inflammation and tumorigenesis [21]. *Enterococcus faecalis* infected macrophages promote DNA double-strand breaks, aneuploidy, and chromosomal instability in murine colonic epithelial cells, which, once transformed, initiate tumor formation in a murine allograft model [22]. The ability of microbes to both directly and indirectly cause DNA damage and genomic instability make the microbiome both a potential risk factor and therapeutic target.

## Conclusions

The densest populations of endogenous microbes are found within the intestines and are in close proximity to the epithelium and underlying mucosal immune system. As a result, the earliest observations linking the microbiota with the hallmarks of cancer have primarily focused on gastric cancers and CRC. Nonetheless, more recent studies have also implicated the microbiota in cancers at distal sites as a potential predictor of successful response to cancer therapy and as a means to augment the efficacy of existing anticancer therapeutics. Furthermore, the well-established link between several viruses and human cancers (i.e., Human papillomavirus and cervical, genital, anal, and oral cancers; Epstein-Barr virus and lymphomas; hepatitis C virus and hepatocellular carcinoma; Kaposi’s sarcoma–associated herpesvirus and Kaposi’s sarcoma) provides a strong rationale to investigate the role of nonbacterial members of the microbiota (virus, fungi, and archaea) in modulating the hallmark capabilities and cancer development. Finally, the cancer microenvironment itself can enhance the procarcinogenic activities of the microbiota [23], which further demonstrates the importance of the crosstalk between host and microbe in modulating cancer progression. In summary, because of the extensive capacity of the microbiota to influence many hallmarks of cancer, treatment for a variety of cancers may soon involve personalized medicine targeting the microbiota.
